# Fundamental Understanding and Construction of Solid‐State Li−Air Batteries

**DOI:** 10.1002/smsc.202200005

**Published:** 2022-03-23

**Authors:** Huan-Feng Wang, Xiao-Xue Wang, Fei Li, Ji-Jing Xu

**Affiliations:** ^1^ College of Chemical and Food Zhengzhou University of Technology Zhengzhou 450044 P. R. China; ^2^ State Key Laboratory of Inorganic Synthesis and Preparative Chemistry College of Chemistry Jilin University Changchun 130012 P. R. China; ^3^ International Center of Future Science Jilin University Changchun 130012 P. R. China

**Keywords:** battery construction, interface regulation between electrodes and electrolyte, solid electrolytes, solid-state Li−air batteries

## Abstract

Nonaqueous Li−air batteries with ultrahigh theoretical energy density have attracted much attention in the development of clean energy technology. However, a series of safety challenges including the flammable, volatile organic liquid electrolyte, together with the electrolyte decomposition have greatly hindered their practical development. Solid‐state electrolytes with superior mechanical strength, good chemical stability under open‐air system, wide electrochemical window, nonflammable properties provide a feasible strategy to overcome the safety issues and achieve a stable, applicable Li−air battery system. In this article, a comprehensive review of solid‐state Li−air batteries is provided. Based on the overall understanding of the necessity of developing a solid‐state Li−air battery and ion migration mechanism in solid electrolytes, the construction strategies of solid‐state Li−air battery including cathode fabrication, Li anode optimization, electrolyte design, and the interface regulation between electrodes and electrolyte are presented. The prospects of solid‐state Li−air batteries are also proposed at the end. It is expected that this review would provide a systematic understanding and theoretical guidance in designing and developing safe, stable, applicable solid‐state Li−air batteries.

## Graphical abstract

This review presents an overview of solid‐state Li−air batteries for next‐generation rechargeable batteries. The progress of solid‐state Li−air batteries including the common solid electrolytes and their ion migration mechanism, the construction strategies of solid‐state Li‐air battery referring to cathode fabrication, Li anode optimization, electrolyte design, the interface regulation between electrodes and electrolyte are comprehensively reviewed.

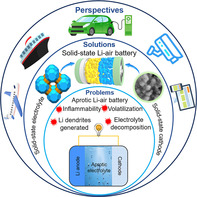

## 1 Introduction

With the industrialization and fast development of the economy in the world, environmental and energy issues have attracted worldwide attention. Nonaqueous Li−air batteries with an ultrahigh theoretical energy density of 3600 Wh kg^−1^ on the basis of the reaction of 2Li + O_2_ = Li_2_O_2_ (*E* ^θ^ = 2.96 V) have attracted much attention in the development of clean energy technology,^[^ ^1^ ^]^ displaying a promising potential achievement. Four types (aqueous, nonaqueous, hybrid, and solid state) of Li−air batteries can be classified based on the utilized electrolyte.^[^ ^2^ ^]^ Among these batteries, nonaqueous Li−air batteries have become one of the focused research for the fascinating specific energy. However, safety and stability challenges caused by the inflammable and volatile nonaqueous electrolyte along with the narrow electrochemical window, poor compatibility with a Li anode, narrow operating temperature range have greatly limited their practical applications.^[^ ^3^ ^]^ The inflammability, volatilization of the commonly utilized organic electrolytes in nonaqueous Li−air batteries would lead to the risk of a battery explosion. Furthermore, Li dendrites generated during the continuous cycles may penetrate the separator and trigger the short circuit. Additionally, the nonaqueous electrolyte is prone to be attacked by the strong nucleophilic intermediates of O_2_^−^ or O_2_^2−^, and the insulated by‐products originated from electrolyte decomposition would increase the overpotential, eventually leading to battery death.

In view of the abovementioned challenges, replacing the aprotic liquid electrolyte with solid electrolyte would provide a promising strategy.^[^ ^4^ ^]^ Solid electrolyte with superior safety, mechanical strength and chemical/electrochemical stability, wide electrochemical window, low‐cost processing, which is favorable to avoiding the electrolyte volatilization and thus sustaining the integrity of the triple‐phase interface, keeping the batteries from combustion or explosion, stopping Li dendrites from penetrating the separator, protecting Li anode from corrosion caused by the harmful reactions between the components from open‐air atmosphere and active Li metal, eliminating the electrolyte decomposition originated from the attack of strong nucleophilic intermediates.^[^ ^5^ ^]^ All these superiorities would greatly guarantee the safety and long cycling stability of Li−air batteries. However, the development of solid‐state Li−air batteries is still in its early stages due to the lack of favorable electrolyte, limited triple‐phase boundaries in the solid cathode, and poor interfacial contact between solid electrolyte and electrodes raised from the inherent solid‐solid phase contact.^[^ ^6^ ^]^

Given all these, in this review, to overcome the challenges of nonaqueous Li−air batteries and achieve practical rechargeable Li−air batteries, the overall understanding of the necessity of solid‐state Li−air battery and ion migration mechanism in solid electrolytes is generally reviewed, then the construction strategies of solid‐state Li−air battery including cathode fabrication, Li anode optimization, electrolyte design, and the interface regulation between electrodes and electrolyte are presented. The prospects of solid‐state Li−air batteries including the exploration of preferable battery materials, battery system, and systematic mechanism study are also proposed at the end. It is expected that this review would provide a systematical understanding and theoretical guidance in designing and developing safe, stable, applicable solid‐state Li−air batteries.

## 2 Necessity of Developing Solid‐State Li−Air Battery

As one of the core components, electrolyte plays a great role in high‐performance Li−air batteries, and the overall battery performance is synthetically affected by each battery component. A large amount of studies have been reported on building highly efficient cathode catalysts, anode protection strategies as well as developing novel electrolytes, and much progress has been achieved.^[^ ^7^ ^]^ However, the stability and safety issues are the two biggest challenges that existed in the commonly studied Li−air battery with organic liquid electrolytes. A series of side reactions of nonaqueous electrolytes, active cathode against the discharge intermediate O_2_^−^ under high potentials, and anode display negative impacts on the stability of batteries. Most importantly, the safety issues caused by the flammable, volatile organic liquid electrolyte are inevitable. The major problems of the commonly studied nonaqueous Li−air battery and the necessity and inevitability of developing solid‐state Li−air battery are illustrated in this section.

### 2.1 Challenges of Nonaqueous Li−Air Batteries

#### 2.1.1 Electrolyte Volatilization

As the cathode active substance of oxygen is directly from the atmosphere, the open working atmosphere of the Li−air battery can hardly inhibit the volatilization of the commonly utilized organic electrolyte. The maintainer of the three‐phase interface plays a key role in superior cycling performance and battery capacity. The gradual electrolyte loss would destroy the integrated solid‐liquid‐gas interface, which is served as the key reaction place of the Li−air battery, leading to the battery failure, more importantly, resulting in a battery explosion. In addition, the inflammability, leakage of organic liquid electrolytes would also lead to serious safety issues.^[^ ^8^ ^]^

#### 2.1.2 Electrolyte Decomposition

Due to the strong oxidizing, nucleophilic discharge intermediates of O_2_^−^or O_2_^2−^ in nonaqueous Li−air batteries, which is prone to react with nonaqueous electrolyte, generating a series of irreversible side‐products such as Li_2_CO_3_, HCOOLi, and CH_3_COOLi. The accumulated side‐products on the cathode would inevitably result in the cathode blocking, which greatly reduces the cycling performance of the Li−air battery and leading to inferior cycling stability.^[^ ^9^ ^]^

#### 2.1.3 Anode Corrosion

Due to the open working environment of the Li−air battery, H_2_O and CO_2_ would shuttle into the inner battery, resulting in a series of side reactions with electrolyte, Li metal anode, and the discharge products. Furthermore, Li anode is also vulnerable to the attack of electrolyte solvents, additives, discharge intermediates the accumulated Li consumption would give birth to a thick insulated solid electrolyte interface (SEI) layer.^[^ ^10^ ^]^ Accordingly, the anode corrosion would result in poor security and short cycle life of Li−air batteries.

#### 2.1.4 Large Concentration Polarization at High Current Density

Due to the large polarization when the battery is charged at a high current density influenced by the sluggish kinetics of oxygen reduction reaction and oxygen evolution reaction, along with the low diffusion rate of O_2_ and Li ions in organic electrolyte and pore channels, the Li−air battery can only work at a small current density, resulting in the inferior rate capability, sharply decreased cycling performance and discharge voltage of Li−air battery.[^9^, ^11^]

### 2.2 Necessity of Developing Solid‐State Li−Air Battery

Based on the abovementioned challenges of nonaqueous Li−air batteries, exploring a favorable electrolyte with superior safety, nonflammability, mechanical strength, chemical/electrochemical stability, wide electrochemical window, is essential. The superiority of solid‐state Li−air batteries with safety and chemical stability under higher charging potential provides a promising strategy to solve the existed problems fundamentally and thus build a high‐performance Li−air battery with superior stability, long cycling life, and superior safety.

Given the above, it is necessary to design a favorable solid‐state Li−air battery to overcome the intractable problems in nonaqueous Li−air batteries, in which the solid electrolytes possess the superior thermal, chemical, and electrochemical stability for high electrochemical reaction kinetics; wide electrochemical window for highly stable electrolyte; favorable solid–solid interface with low interface impedance for high mass transfer, high ion transference number, favorable ionic conductivity in the suitable temperature range; as well as the high mechanical strength for preventing Li dendrite penetration and achieving a thin, large‐area film with low ionic impedance, low‐cost processing. In view of the aforementioned advantages, ideal solid‐state Li−air batteries are beneficial to achieving safe, practical batteries, although some problems such as the relatively large interface impedance between electrodes and solid electrolyte, the stability issues toward the complex air constituents, the questionable compatible contact between electrolyte and Li anode in open‐air operating system are still required to be carefully considered.

## 3 Progress and Development of Solid‐State Li−Air Batteries

**Figure 1:**
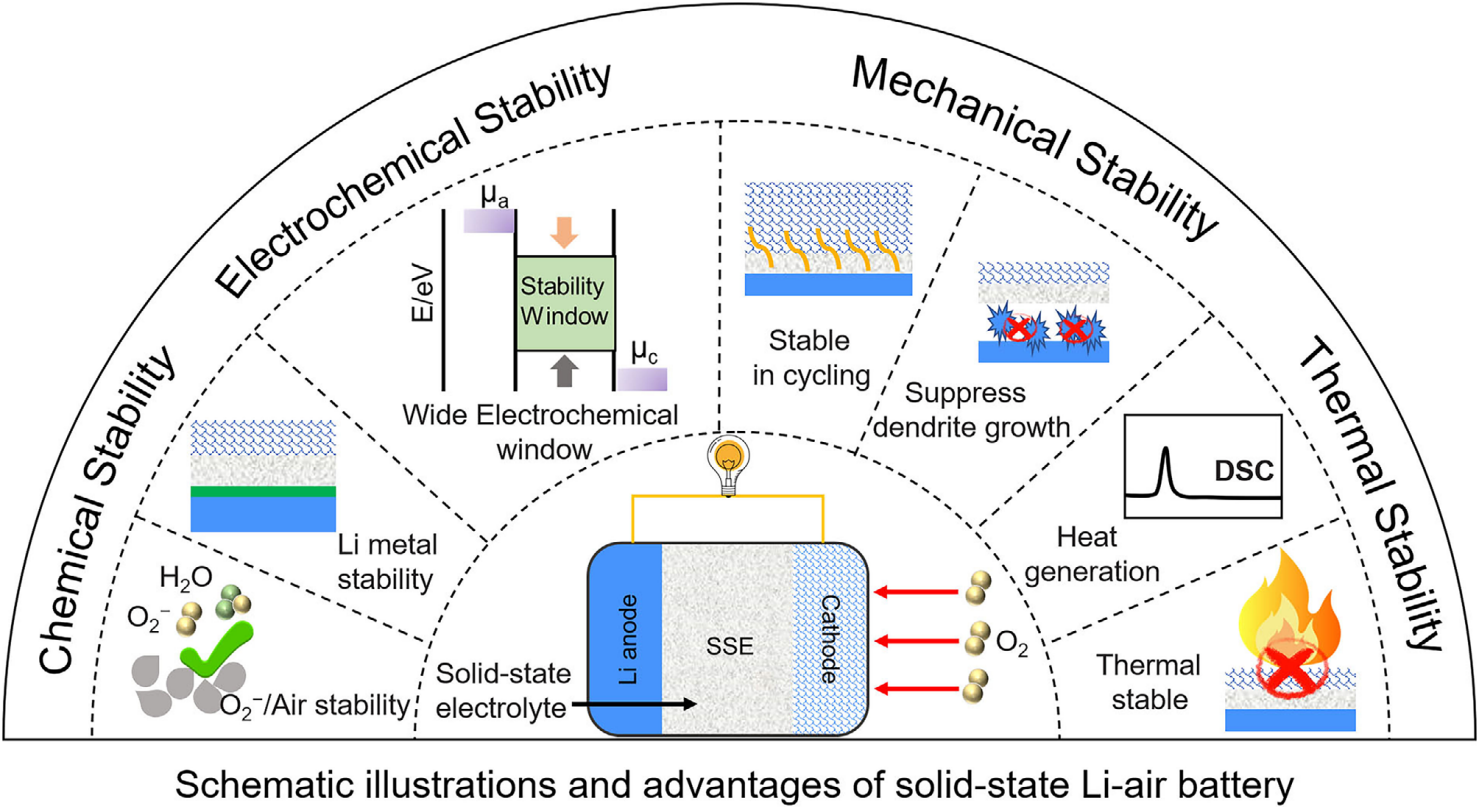
Schematic illustrations and advantages of solid‐state Li–air battery.

The first solid‐state Li−air battery with a similar structure to a solid‐state fuel cell was constructed by Sammells and Semkow in 1987, by adopting La_0.89_Sr_0.10_MnO_3_ as the cathode, solid ZrO_2_ as the electrolyte, the immersing FeSi_2_Li_*x*_ into ternary molten salts as the anode.^[^ ^12^ ^]^ Its theoretical discharge capacity is up to 4266 Wh kg^−1^. Due to the high operating temperature of 600–850 °C (under which the O^2−^ is prone to pass through the solid ZrO_2_ and react with Li ions to ensure the higher discharge capacity) and the complex battery components, the development of this solid‐state Li−air battery has been impeded. Then Li−air batteries with Li−ion conductive solid electrolytes are developed to achieve the low interface impedance, which is composed of a porous air cathode, Li anode, and solid‐state electrolyte, similar to the nonaqueous Li−air batteries except for the aggregating status of the electrolyte (**Figure** **1**). Kumar et al. first constructed a NASICON‐structural‐electrolyte‐based solid‐state Li−air battery, with the composition of 18.5Li_2_O:6.07Al_2_O_3_:37.05GeO_2_:37.05P_2_O_5_ (LAGP) as the electrolyte, Li metal as the anode, porous carbonaceous materials as the cathode, incorporated poly(ethylene oxide) (PEO) with a Li salt composites as buffer layers to achieve low interface impedance.^[^ ^13^ ^]^ With this favorable construction, a Li ionic conductivity of 10^−4^ S cm^−1^ can be achieved when the operation temperature is up to 60 °C. Still later, a Li_7_La_3_Zr_2_O_12_ (LLZO) electrolyte‐based solid‐state Li−air battery without incorporating layer coating was developed by Sakamoto and coauthors.^[^ ^14^ ^]^ Taking advantage of this simple battery configuration, an amazing lower interfacial resistance of 2 Ω cm^2^ even comparable to the liquid electrolyte was achieved. With the increased fundamental cognition of Li−air batteries, various solid electrolytes have been developed in recent years. In this section, the progress of solid‐state Li−air batteries referred to the general mechanism of ion migration, commonly studied solid electrolytes, construction strategies of solid‐state Li−air batteries are discussed, respectively.

### 3.1 Mechanism of Ion Migration

**Figure 2:**
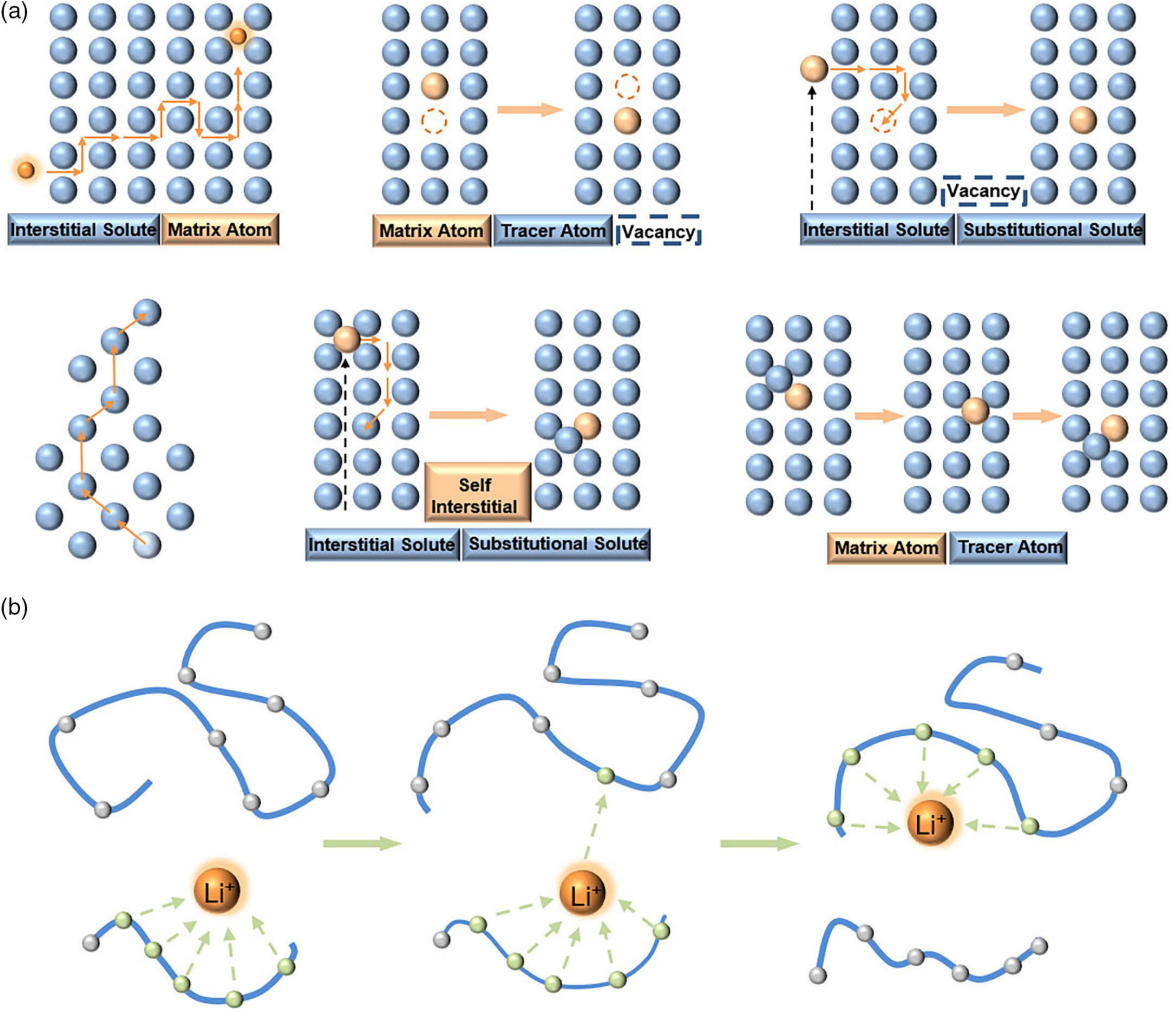
General mechanisms of ion migration in: a) inorganic solid electrolytes and b) polymer electrolytes, respectively.

The ion migration in solid electrolytes plays a decisive role in the energy efficiency and output power of solid‐state Li−air batteries.^[^ ^15^ ^]^ The migration mechanisms for ions crossing through the solid electrolytes are mainly determined by the located sites and the energy, which can be concluded as the following aspects: For the inorganic crystalline electrolytes, the ions from one site can migrate into an adjacent vacant site through vacancy diffusion, the ions transfer through direct interstitial mechanism between the incomplete occupied sites or correlated interstitial mechanism by displacing the adjoining lattice to the neighboring site with the migrating interstitial ion (**Figure** **2**a).^[^ ^16^ ^]^ For the polymer electrolytes, the ions migration is achieved through the consecutive coordination of mobile ions and polar groups (Figure 2b).^[^ ^17^ ^]^

The key descriptor for ion transport is indicated by a function of *σ,* which is defined as *σ*(T) = *qnu *=*σ*_0_exp[−*E*_a_/*k*_B_ *T*] for inorganic crystalline electrolytes, in which *q* indicates the product charge, *n* is the product concentration, and *u* presents mobility of charge carriers in a solid crystalline electrolyte; *σ*_0_ is the pre‐exponential factor that determines the intrinsic carrier density of the material, *m* is generally equal to −1, *k*_B_ indicates the Boltzmann constant, *T* is the temperature, and *E*_a_ means characteristic activation energy of ion conduction including the energy for the formation of mobile defects and the energy barrier for their migration. The ionic conductivity of a solid polymer electrolyte is described by the following equation *σ*(T) = *σ*_0_ *T* ^1/2^ exp[−*E*_a_/*R*(*T*−*T*_0_)], in which *σ*(T) indicates the ionic conductivity of the polymer solid electrolyte at a certain temperature, *σ*_0_ is the pre‐exponential factor which determines the intrinsic carrier density of the material, *E*_a_ is the activation energy of the material, *R* means the ideal gas constant, and *T* is the reference temperature which is 10–50 K lower than the glass transition temperature of the polymer. When the temperature is higher than the glass transition temperature of the polymer, Li ions are prone to migrate along with the irregular movement of the chain segment of the polymer electrolyte, and thus lowering the glass transition temperature of the polymer is beneficial to improving the ionic conductivity of the polymer. However, due to the complexity of the polymer system and the shortage of systematic study on the crystal structures, the transport mechanism of Li‐ion in polymer solid electrolyte is still inadequate at present, therefore, a more detailed, systematic study is necessary to be carried out to elaborate the transport mechanism of Li ions.

Furthermore, understanding the mechanism of ions migration from varying scales is of guiding significance to achieve high ionic conductivity for tackling the major challenge of high interfacial resistance in solid‐state Li−air batteries. Recently, Famprikis et al. summarized and discussed the mechanism of ion transport combined with the major associated techniques in an inorganic solid electrolyte at different length scales including the atomic scale, micro‐ and mesoscopic scales, macroscopic scale, device scale, which is valuable to deepen the study of solid‐state battery technology from microsystem level to the macroscopic perspective.^[^ ^16^ ^]^ Accordingly, with the development of solid‐state Li−air battery, systematic, in‐depth research with the combination of theoretical calculation and advanced characterization techniques is expected to achieve a definite cognition of ion‐transport mechanisms in the solid electrolyte.

### 3.2 Solid Electrolyte in Li−Air Battery

According to the abovementioned mechanisms of ionic conductivity, the commonly studied solid electrolytes can be classified into three categories: inorganic solid electrolytes, polymer solid electrolytes, and composite solid electrolytes.

#### 3.2.1 Inorganic Solid Electrolyte

**Figure 3:**
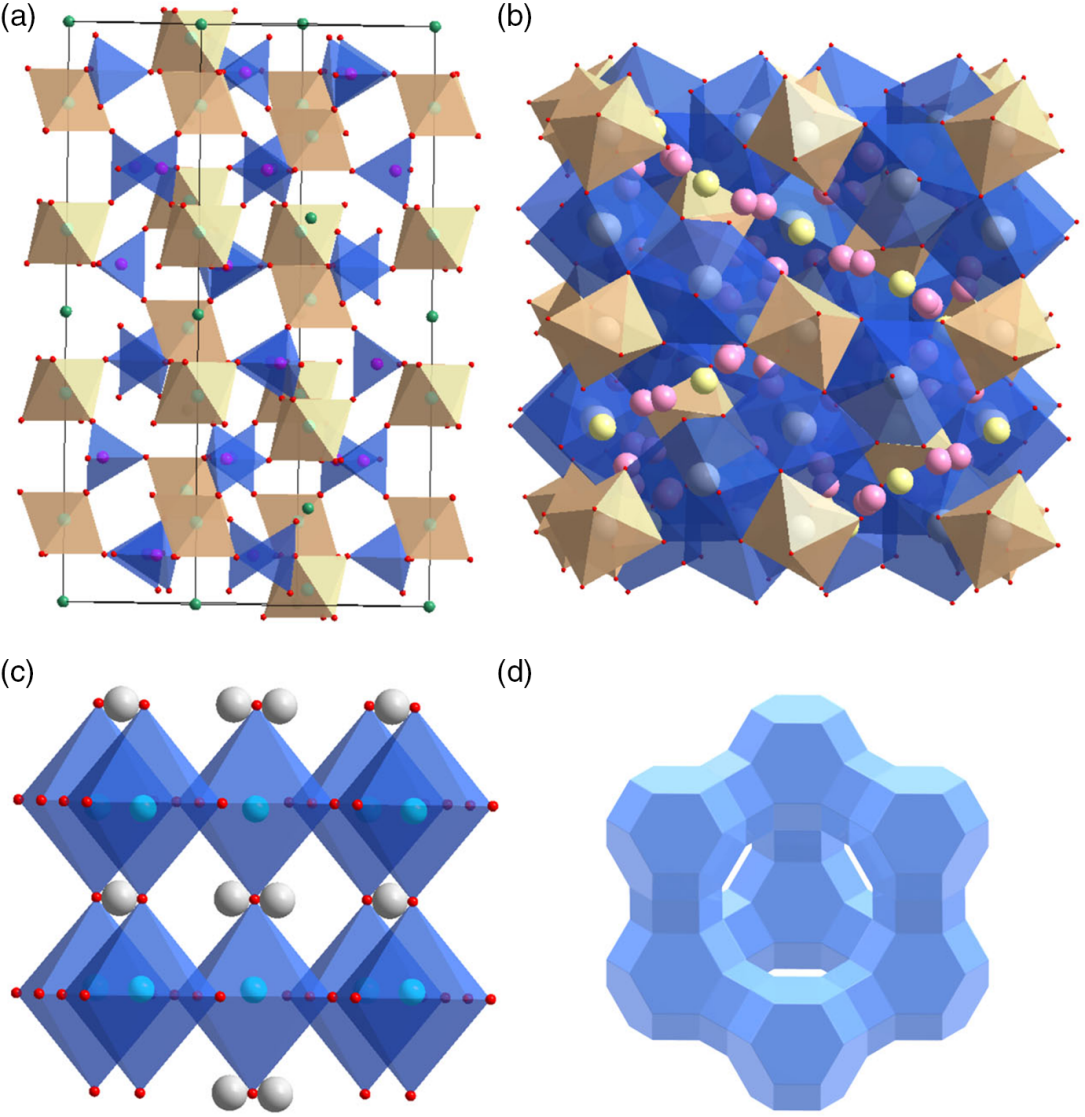
Several typical crystal structures of inorganic solid electrolytes. a) NASICON‐type LAGP. b) Garnet‐type LLZO. c) Perovskite‐type LLTO. d) Zeolite X.

The utilized inorganic solid electrolytes can be further classified as inorganic oxides including sodium superionic conductor NASICON, garnet, perovskite and antiperovskite, zeolites, as well as inorganic sulfides such as Li_2_S‐P_2_S_5_ system and thio‐LISICON. Among these inorganic solid electrolytes, inorganic sulfides are superior in high ionic conductivity, wide electrochemical window, good compatibility with Li metal anode,^[^ ^18^ ^]^ but with the fatal drawback of the generation of toxic H_2_S gas due to their sensitivity to H_2_O in air,^[^ ^19^ ^]^ and the preparation, storage, use of such electrolyte under inert atmosphere make it inconvenient to be adopted in the open air. Relatively, inorganic oxides exhibit favorable Li‐ions conductivity, highly chemical stability in the open operating atmosphere, wide electrochemical window, fine mechanical strength are considered to be promising in developing practical solid‐state Li−air batteries. In this section, we mainly focus on inorganic oxides. Several typical crystal structures of solid inorganic electrolytes are displayed in **Figure** **3**, which will be illustrated in detail, respectively.

NASICON‐type solid electrolyte displays a 3D network with a general formula of AM(PO_4_)_3_, along with rhombic unit cell and space group *R*3*c*. In the NASICON structure, A site is commonly occupied by the base metal Li, Na, or K, while M site is usually occupied by Ge, Zr, or Ti.^[^ ^20^ ^]^ According to the structured distortion and the radius of the alkali metal ions, the alkali metal ions would account for different sites, combining with the ion migration channels to form the ion conduction network of NASICON structure for high ionic conductivity. Li‐ion‐conducting electrolyte of Li_1+*x*_M_*x*_Ti_2−*x*_(PO_4_)_3_ can be achieved by substituting Na ions with Li‐ions. The ionic conductivity can be further improved by the substitution of other metals, among which Al substitution is proved to be most effective due to the bottleneck for Li‐ion conductivity being widened by the doping Al^3+^.^[^ ^21^ ^]^ Therefore, one most commonly utilized NASICON structure solid electrolytes with Li titanium phosphate systems of Li_1+*x*_Al_*x*_Ti_2−*x*_(PO_4_)_3_ (LATP) exhibit comparatively higher total ion conduction of over 10^−4^ S cm^−1^ at room temperature. However, Ti^4+^ is prone to be reduced to Ti^3+^ by Li metal when in direct contact with Li metal, which can be solved by the addition of an interlayer such as a polymer electrolyte.^[^ ^22^ ^]^ Another solution is the utilization of stable Ge^4+^ doping,^[^ ^23^ ^]^ and the obtained Li_1+*x*_Al_*y*_Ge_2−*y*_(PO_4_)_3_ (LAGP) is proved to be more stable toward Li metal, as well as a wide electrochemical window. Both LATP and LAGP display superior stability in the open air, which is promising in practical Li−air battery.

A new class of Li‐ion conductor, garnet‐structure Li‐ion conductor has a general formula of LiA_3_B_2_O_12_, in which Li^−^ion occupies the four coordination sites. The garnet‐structural solid electrolyte is superior in thermal and chemical stability, low activation energy, and mechanical strength. The ionic conductivity can further be enhanced by regulating the valence states of A^3+^ and B^6+^ to introduce more Li ions. Murugan et al. first reported garnet‐type LLZO ceramics with the highest Li content in 2007.^[^ ^24^ ^]^ The sheet‐like LLZO owns an ionic conductivity of 7.74 × 10^−4^ S cm^−1^(with a thickness of 0.18 cm) at room temperature and low activation energy of 0.34 eV, which is considered to be the highest ionic conductivity in garnet structure ionic conductor. However, the sensitivity to H_2_O and CO_2_, high interfacial resistance with Li anode are considered to be two main drawbacks of the garnet‐structure solid electrolyte.^[^ ^25^ ^]^ The introduction of a metal, metal oxide, or polymer‐based intermediate layer between the solid electrolyte and Li anode would be promising in improving the adhesion of garnet electrolyte with Li anode, contributing to the low interfacial resistance.^[^ ^26^ ^]^

The perovskite‐type solid electrolyte is a class of crystalline material with a general formula of ABO_3_ and cubic unit cell with the space group of Pm/3m, in which A indicates Ca, Sr, La, and B indicates Al, Ti. Li‐ion can be introduced into perovskite by the elements doping to achieve a favorable Li‐ion conductor of Li_3*x*_La_2/3−*x*£1/3−2*x*_TiO_3_ (LLTO). The type of A‐site cations, Li ions, and vacancy concentration, as well as the interaction between vacancy and Li‐ions affect the ionic conductivity of LLTO. The Li‐ions are prone to migrate along the A‐site vacancy to the adjacent vacancy through the bottleneck. Therefore, the ionic conductivity of the perovskite ion conductor can be improved by controlling the concentration of vacancy and Li‐ions, adopting larger radius rare‐earth or alkaline‐earth ions to replace La^3+^ in LLTO. For instance, the ionic conductivity of the LLTO system can be improved by replacing La^3+^ with a larger ionic radius Sr^2+^ in LLTO, and a crystal conductivity of 1.5 × 10^−3^ S cm^−1^ at room temperature can be achieved. However, some challenges such as the low total conductivity of LLTO due to the high grain boundary resistance, the unstable issues LLTO towards Li metal because of the existence of oxidizing Ti^4+^ in LLTO still need to be considered. By contrast, antiperovskite‐type ion conductor owns a general formula of Li_3_OA (A can be Cl, Br), in which Li ions are located at the vertices of the regular octahedron, while O^2−^ resides in the center of the regular octahedron, and A^−^ is situated in the center of the cube. With the advantages of the anti‐perovskite solid electrolyte including low activation energy for favorable Li‐ion migration, wide electrochemical working window for stable contact with Li metal, environment‐friendly, and low‐temperature preparation, this solid electrolyte displays application prospect in solid‐state Li−air battery.^[^ ^27^ ^]^

**Figure 4:**
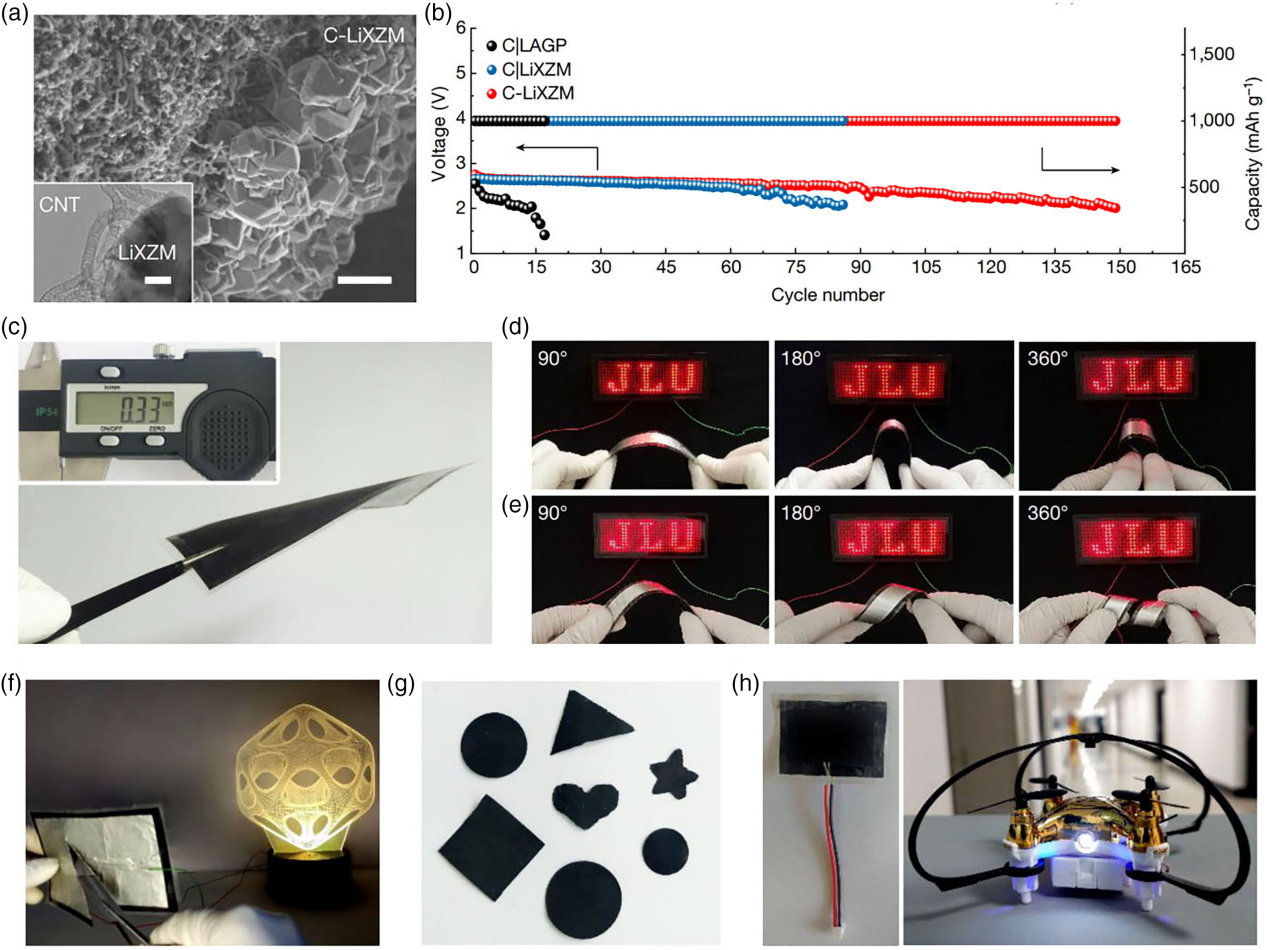
a) Scanning electron microscopy (SEM) of the integrated structure of carbon nanotube cathode‐solid zeolite electrolyte (C‐LiXZM) and transmission electron microscopy images of the cathode/electrolyte interface (inset). b) The cycle life comparison of Li−air batteries with different solid electrolytes. c) The optical photograph of C‐LiXZM‐based solid‐state Li−air battery with a thickness of merely 0.33 mm. d) The flexibility tests of the integrated solid‐state Li−air battery based on C‐LiXZM under different bending and e) torsion conditions. f) Safety and environmental adaptability of C‐LiXZM‐based solid‐state Li−air batteries. g) Superior shape modifiability of C‐LiXZM‐based solid‐state Li−air battery. h) The application of the integrated solid‐state Li−air batteries based on C‐LiXZM in powering an unmanned aerial vehicle. Reproduced with permission.^[^ ^29^ ^]^ Copyright 2021, Nature Publishing Group.

Zeolites are a family of crystalline silicate or aluminosilicate with the connection of silicon‐oxygen tetrahedrons or aluminotetrahedrons joined by oxygen bridge bonds, which is typical of an open framework with orderly distributed micropores.^[^ ^28^ ^]^ Taking advantage of the unique microporous structure and the continuous ion‐conduction pathway, a fast ion migration can be achieved among crystals. A novel inorganic electrolyte represented by zeolite for assembled Li−air batteries was developed by Yu, Xu, and coauthors in 2021, which could effectively solve the interface construction issues, the internal Li dendrites, and poor stability toward the air components of the traditional solid electrolyte materials in Li−air batteries, as well as retaining the superior Li‐ionic conductivity.^[^ ^29^ ^]^ Benefiting from the reasonable pore structure and abundant Li‐ions distribution with low activation energy sites, the novel solid electrolyte (LiXZM) displayed a high ionic conductivity of 2.7 × 10^−4^ S cm^−1^, low electronic conductivity of 1.5 × 10^−10 ^S cm^−1^, and superior chemical stability toward both air composition and Li anode. Due to the “electrolyte–electrode” low‐impedance contact intimate interface between the solid electrolyte and both electrodes (**Figure** **4**a), the flexible solid‐state Li−air battery containing the integrated structure of carbon nanotube cathode‐solid zeolite electrolyte (C‐LiXZM) displayed an ultrahigh discharge capacity of 12 020 mAh g^−1^ and a long cycle life of 149 cycles at a current density of 500 mA g^−1^ and a fixed capacity of 1000 mAh g^−1^ under the air atmosphere, which was much higher than that of NASICON LAGP‐based solid‐state Li−air battery of 13 cycles (Figure 4b). At the same time, the battery exhibited excellent flexibility, high safety, superior shape modifiability, and good environmental adaptability (Figure 4c–h). Furthermore, this solid zeolite‐based electrolyte showed a broad application prospect and promotes the innovation and development of solid electrolytes and related solid‐state energy storage batteries, which is also expected to be extended to other novel energy storage systems such as Li‐ion battery, Na−ion battery, Mg−ion battery, Na−air battery.

To sum up, among the inorganic solid electrolytes, inorganic sulfides possess high ionic conductivity, wide electrochemical window, good compatibility with Li anode, but they are unstable in open‐air atmosphere and would generate the toxic H_2_S, which seem inconvenient to apply in Li−air batteries compared with the inorganic oxide solid electrolytes. Among the inorganic oxide solid electrolytes, NASICON and garnet solid electrolytes exhibit relatively high conductivity, while perovskite and antiperovskite solid electrolytes display lower conductivity, but instability toward air and Li metal for some garnet and NASICON solid electrolytes are still needed to be seriously considered. Zeolites with good ion conductivity and high chemical stability toward both air components and Li anode provide a promising prospect in solid‐state Li−air batteries.

#### 3.2.2 Polymer Solid Electrolyte

As mentioned earlier, inorganic ceramic solid electrolytes with good ionic conductivity, wide electrochemical window, highly safe performance, wide application scope play a key role in the development of solid‐state Li−air batteries. However, some challenges including the instability towards H_2_O, CO_2_ under air atmosphere, poor contact with the electrode, high cost, and difficult process still hinder their large‐scale application process in practical Li−air batteries. By contrast, polymer solid electrolytes which is superior in machinability and flexibility display unique advantages and displays the inhibiting ability of Li dendrite formation to some extent, has gained important research direction in the solid electrolyte.

Commonly, the solid polymer electrolyte can be obtained by dissolving Li salts such as Li bis(trifluoromethanesulfonyl)imide (LiTFSI), Li trifluoropotassium sulfonate (LiCF_3_SO_3_), Li bisfluorosulfonimide (LiFSI) into the polymer chain, i.e., PEO, polyacrylic acid (PAA), polymethyl methacrylate (PMMA), displaying excellent mechanical strength. PEO, as the first utilized polymer solid electrolyte, with a series of advantages such as excellent dimensional stability, good mechanical properties has been widely studied in Li–metal batteries. Usually, crystalline PEO under low temperature displays low ionic conductivity, while amorphous PEO exhibits higher ionic conductivity but inferior dimensional stability when the temperature surpasses the glass transition temperature.^[^ ^30^ ^]^ Even so, in comparison with the high ionic conductivity of the inorganic ceramic solid electrolytes, the ionic conductivity of PEO‐based electrolytes is still low. A series of strategies in improving the Li‐ion transference number such as the addition of Li salts containing large anionic groups, developing quasi‐solid gel polymer electrolytes by adding inorganic filler and plasticizer have been developed. In addition, polymer blending can also provide an effective strategy to lower the crystallinity of polymer, which is referred to in the following discussed composite solid electrolyte. Although PEO is considered to be the most widely studied organic polymer electrolyte, it is prone to be oxidized by the discharge product of Li−air batteries. Harding et al. studied the stability of PEO‐based Li−air batteries, the results demonstrated that PEO was unstable and was prone to occur the auto‐oxidation in an oxidized environment, which seriously influenced the cycle efficiency.^[^ ^31^ ^]^ Amanchukwu et al. studied the structural unit of various polymers and their stability to Li_2_O_2_ by combining the experiment results with various characterization technologies.^[^ ^32^ ^]^ The results indicated that polyacrylonitrile (PAN) containing electrophilic groups is prone to be attacked by Li_2_O_2_, polyvinyl chloride (PVC), polyvinylidene fluoride (PVDF), polyvinylidene fluoride hexafluoropropylene (PVDF‐HFP) containing halogens and adjacent α, β‐H would be in an electron‐deficient state due to the hyperconjugation effect and react with Li_2_O_2_, while polytetrafluoroethylene (PTFE), perfluorosulfonic acid resin (Nafion), PMMA exhibited superior stability toward O_2_^−^. The research provided great guidance for selecting the ideal solid polymer electrolyte for Li−air battery. Replacing some of the unstable H atoms in the polymer chains with other functional groups would be an alternative strategy to inhibit the nucleophilic attack during discharging and the addition of antioxidants is favorable to inhibiting the degeneration of polymer electrolytes.^[^ ^33^ ^]^

Accordingly, although several drawbacks existed in polymer solid electrolytes such as the low Li ionic conductivity caused by the crystallization, the decomposition of the polymer matrixes limit their application under open operating atmosphere to some extent, the high tolerance to the battery volume change, superior process ability of the polymer solid electrolytes make them promising in developing flexible solid Li−air batteries.

#### 3.2.3 Composite Solid Electrolyte

According to the previous statement, poor compatibility between electrolyte and electrode but superior ionic conductivity exist in the inorganic ceramic solid electrolyte, low ionic conductivity but excellent dimensional stability, good mechanical properties are consisted in single polymer solid electrolyte at room temperature. To achieve an ideal solid electrolyte, the construction of composite electrolyte by taking advantage of the synergistic effect of the different types of electrolyte composition and compensating for each drawback is surely regarded as an effective means.^[^ ^34^ ^]^ The commonly studied composite solid electrolyte can be divided into the following two main categories: inorganic–organic composite solid electrolyte, solid–liquid composite electrolyte.

According to the differences in composition and content of the filler and the matrix, the commonly studied solid inorganic–organic composite electrolyte can be classified into three categories as ceramic‐in‐polymer, polymer‐in‐ceramic, and intermediate. For the ceramic‐in‐polymer structural composite solid electrolyte, the polymer is adopted as the matrix and inorganic ceramic as the filler is dispersed in polymers, while polymer as the filler is dispersed in the inorganic ceramic matrix for polymer‐in‐ceramic structure, and a roughly comparable content of polymer and ceramic is considered to be intermediate. Yi et al. reported a composite solid electrolyte with a mass ratio of 1:1 composition of polymer solid electrolyte polystyrene methyl methacrylate (PMS) and NASICON solid electrolyte LAGP.^[^ ^35^ ^]^ The obtained composite electrolyte displays both high ion transference number and superior flexibility. The Li−O_2_ battery based on this composite electrolyte displayed a stable cycle performance of 350 cycles. Pan et al. introduced a polymer‐in‐ceramic structured composite electrolyte membrane composed of 89.6 wt%Li_6.6_La_3_Zr_1.6_Ta_0.4_O_12_ (LLZTO) garnet matrix and a supramolecular filler of crown ether Li salt complex into a solid‐state Li−air battery.^[^ ^36^ ^]^ Benefiting from the combined advantages of each respective component, the as‐prepared composite membrane displayed a high ionic conductivity of 6.92 × 10^−4^ S cm^−1^ at 20 °C, and optimized interfacial compatibility between solid electrolyte and Li anode, furthermore, the I_3_^−^/I^−^ redox mediator in the supramolecular filler played an effective role in reducing the discharge–charge voltage gap (800 mV smaller than LLZTO membrane) and achieving an enhanced battery cycling stability of 21 stable cycles without any capacity fading compared to merely 2 cycles for the single LLZTO membrane. In addition, solid composite electrolytes with high ionic conductivity and superior electrochemical stability are also prepared by doping the passive ceramic filler with solid polymer electrolytes, in which the introduction of passive ceramic filler such as Al_2_O_3_, SiO_2_, TiO_2_ would lower the crystallinity in the polymer and thus improve the ionic conductivity.^[^ ^37^ ^]^ The passive SiO_2_ fillers were doped with PEO by Cui's group.^[^ ^38^ ^]^ Benefiting from the improved interactions, good ionic conductivity of 4.4 × 10^−5^ S cm^−1^ at 30 °C), and a wide electrochemical window up to 5.5 V (versus Li^+^/Li) without obvious decomposition was achieved.

**Figure 5:**
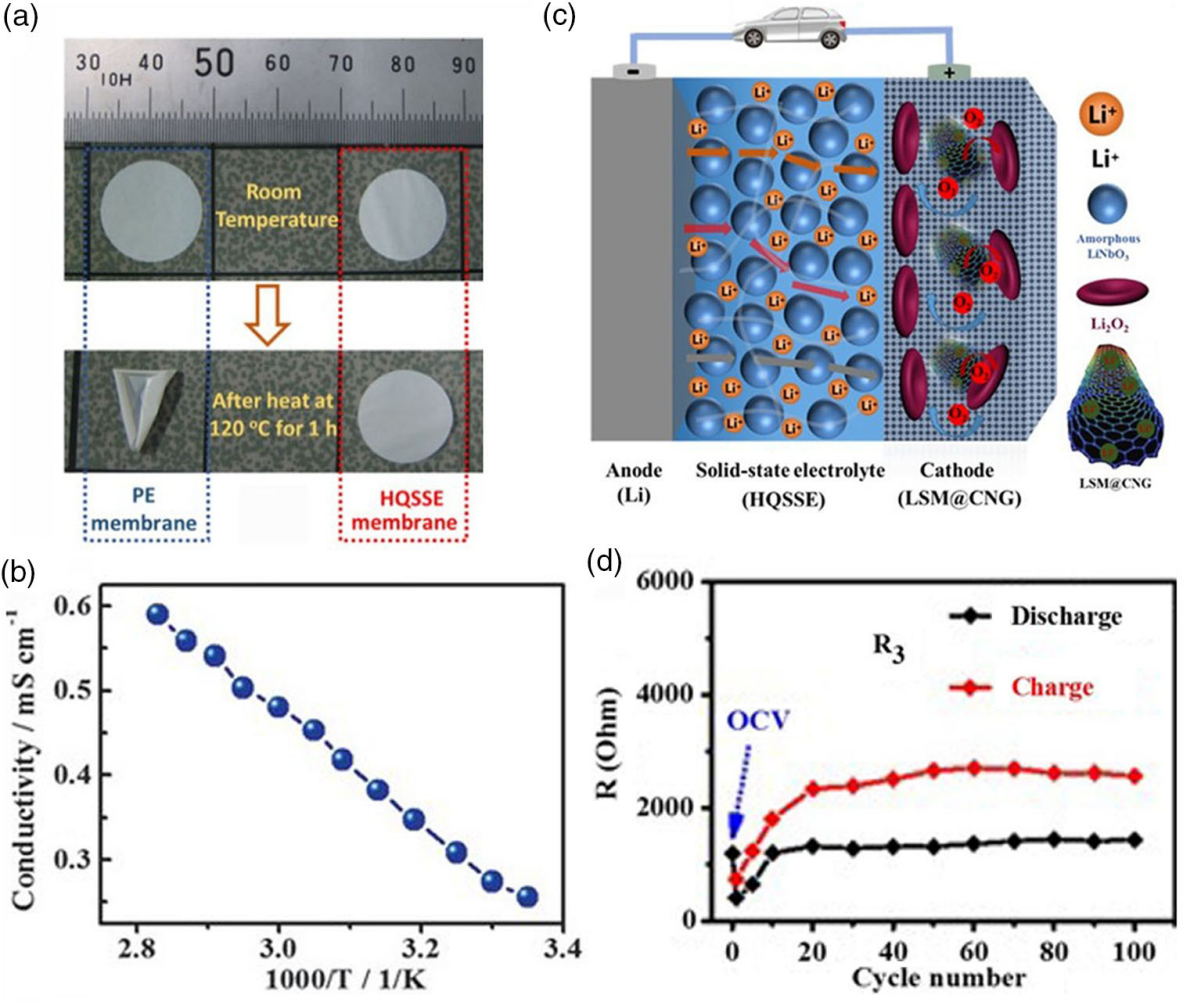
a) The shrinkage comparison of PE and HQSSE membranes at 120 °C for 1 h. b) The ionic conductivity of HQSSE versus temperature represented with Arrhenius plots. c) The proposed HQSSE‐based solid‐state Li−O_2_ battery with Li salt‐modified single‐walled carbon nanotubes and an ionic‐liquid‐based cross‐linked network gel (LSM@CNG) as a cathode. d) The corresponding interfacial resistance obtained from the Equivalent circuit fitting results. Reproduced with permission.^[^ ^42^ ^]^ Copyright 2016, Wiley‐VCH.

Additionally, the solid–liquid composite electrolyte is composed of one or more nonaqueous liquid electrolyte and at least one type of solid matrix, such as the gel polymer electrolyte, ionogel electrolyte, that is, the quasi‐solid electrolyte. Gel polymer electrolyte is considered to be a typical solid–liquid composite electrolyte with the composition of aprotic electrolytes and solid matrixes, in which the solid matrixes are swollen with the aprotic electrolytes. The obtained gel polymer electrolyte combines the favorable mechanical property of a swollen polymer framework with the high ionic conductivity and good interfacial properties of an aprotic electrolyte or the other solid electrolyte.^[^ ^39^ ^]^ PVDF‐HFP, PAN, PMMA, PVC are commonly utilized as the matrix, and dimethyl carbonate, ethylene carbonate is adopted as the swelling solution, respectively. Benefiting from the quasi‐liquid properties of the swelling polymer matrix, the Li‐ionic conductivity can be greatly enhanced. The first quasi‐solid gel polymer electrolyte‐based Li–air battery was developed by Abraham, which was constructed with PAN.^[^ ^40^ ^]^ Mohamed et al. designed natural rubber‐based gel polymer electrolytes for Li−air batteries by adopting natural rubber as the polymer matrix, LiCF_3_SO_3,_ and ethylene carbonate or propylene carbonate as the plasticizer.^[^ ^41^ ^]^ The ionic conductivity is up to 4.92 × 10^−4^ S cm^−1^, and the battery delivered a high discharge capacity of 61 mAh g^−1^ at room temperature. The utilization of natural rubber polymer provided a direction in developing environmentally friendly, safety, low‐cost Li–air batteries. Zhou's group constructed a hybrid quasi‐solid state electrolyte (HQSSE) by mixing the polymer electrolyte of PMS with the ceramic electrolyte of amorphous LiNbO_3_ in aprotic tetrahydrofuran together with the polyethylene (PE) supporter.^[^ ^42^ ^]^ The as‐prepared quasi‐solid electrolyte displayed superior thermal stability without shrinkage or degradation even at 120 °C (**Figure** **5**a) and high ionic conductivity (Figure 5b). Benefiting from the stable interfacial resistance between as‐prepared quasi‐solid state electrolyte and oxygen cathode, and its positive effect in suppressing the Li dendrite growth, the Li–O_2_ battery based on HQSSE retained stable discharge/charge processes over 100 cycles (Figure 5c,d). Meng et al. designed a gel polymer electrolyte based on poly(vinylformal)‐based Janus membrane supporting and assembled for Li–O_2_ batteries. The prepared gel polymer electrolyte displayed superior compatibility toward Li anode and carbon cathode, and lower interfacial impedance of electrode/electrolyte.^[^ ^43^ ^]^ The Li–O_2_ batteries based on this gel polymer electrolyte membrane performed a prolonged cycling life of 150 cycles and a discharge capacity of 8634.4 mAh g^−1^. Aside from the abovementioned aprotic electrolytes, as a particular class of nonaqueous liquid electrolyte, ionic liquid at room temperature with negligible vapor pressure for little electrolyte evaporation in an open atmosphere, wide electrochemical window, high chemical/electrochemical as well as thermal stability also shows great potential in developing safe, stable composite solid electrolyte.^[^ ^44^ ^]^ Ionogel electrolyte consists of the ionic liquid at room temperature and solid matrix, which perfectly combines the features of ionic liquid and solid electrolyte. Especially with the positive role of hydrophobic ionic liquid in inhibiting the Li anode corrosion of Li−air batteries in an open environment, the development of ionogel electrolyte is of great value to highly safe, practical solid‐state Li−air batteries. An ionic liquid of 1‐ethyl‐3‐methylimidazolium bis(trifluoromethanesulfonyl)imide (EMITFSI) with different weight ratios was utilized to construct an ionogel composite electrolyte along with the polymer PVDF‐HFP, LiTFSI and applied to quasi‐solid state Li−air battery.^[^ ^45^ ^]^ Due to the lowering crystallinity of polymer matrix and generation of large‐size cross‐linked pores by incorporating EMITFS into the polymer electrolyte, the obtained composite electrolyte displayed an electrochemical stability window of 4.9 V together with a superior ionic conductivity of 4.30 × 10^−3^ S cm^−1^), superior compatibility with Li‐metal anode and air cathode. The assembled quasi‐solid‐state Li−air battery displayed a stable cyclic performance for 20 cycles without capacity fading.

In conclusion, among the above‐mentioned three major types of solid electrolytes, due to the synergistic effect of the different types of the electrolyte composition and compensate for each drawback, the composite electrolyte would be more favorable in developing future solid‐state Li−air battery.

### 3.3 Construction Strategies of Solid‐State Li–Air Battery

Different from Li–O_2_ battery, more complex reaction mechanism of solid‐state Li–air battery occurs under air operating atmosphere due to the complicated components including O_2_, H_2_O, CO_2_, and so on. During discharge, except for the main electrochemical reaction of Li_2_O_2_ generation, side reactions between Li_2_O_2_ and H_2_O, CO_2_ would lead to LiOH and Li_2_CO_3_ formation. During charge, Li_2_O_2_ is initially decomposed which is followed by the removal of the by‐products of Li_2_CO_3_ and LiOH. Therefore, one discharge plateau representing the generation of the discharge products of Li_2_O_2_ and two charge platforms referring to the initial Li_2_O_2_ decomposition and the subsequent removal of Li_2_CO_3_ and LiOH are generally included in solid‐state Li–air battery.[^4^, ^46^] Therefore, to achieve favorable, practical high‐performance solid‐state Li–air batteries, the following key issues referring to air cathode, Li anode, solid electrolyte as well as the regulation of electrolyte/electrode interfaces should be well considered during the design and construction of the battery: 1) Battery component optimization. Excellent chemical, electrochemical, and thermal stability of electrode and electrolyte is of vital importance. The superior chemical and electrochemical stability is capable of ensuring the electrolyte or electrode free from unexpected reactions with other components of the battery to achieve high efficiency and stable cycling performance, while good thermal stability is quite valuable for the safety of the battery. Therefore, a porous cathode with high conductivity, superior electrochemical stability, and high catalytic activity is indispensable to ensure fast core reactions during the charge/discharge process. Robust, chemically stable Li anode should be achieved to restrain the Li dendrite formation and overcome the volume change during Li plating/stripping. Electrolyte with superior ionic conductivity, wide electrochemical window for stability toward both cathode and anode, superior mechanical strength to withstand the penetration of Li dendrites, higher Li‐ion transference number for decreased polarization during the charge and discharge process, is a key factor in determining the internal resistance and the rate performance of the battery, especially for the solid electrolyte; and 2) Electrolyte/electrode interface regulation. Favorable interface with negligible interfacial impedance and good compatibility between electrode and electrolytes is essential to achieving a practical Li−air battery.

#### 3.3.1 Cathode Fabrication

**Figure 6:**
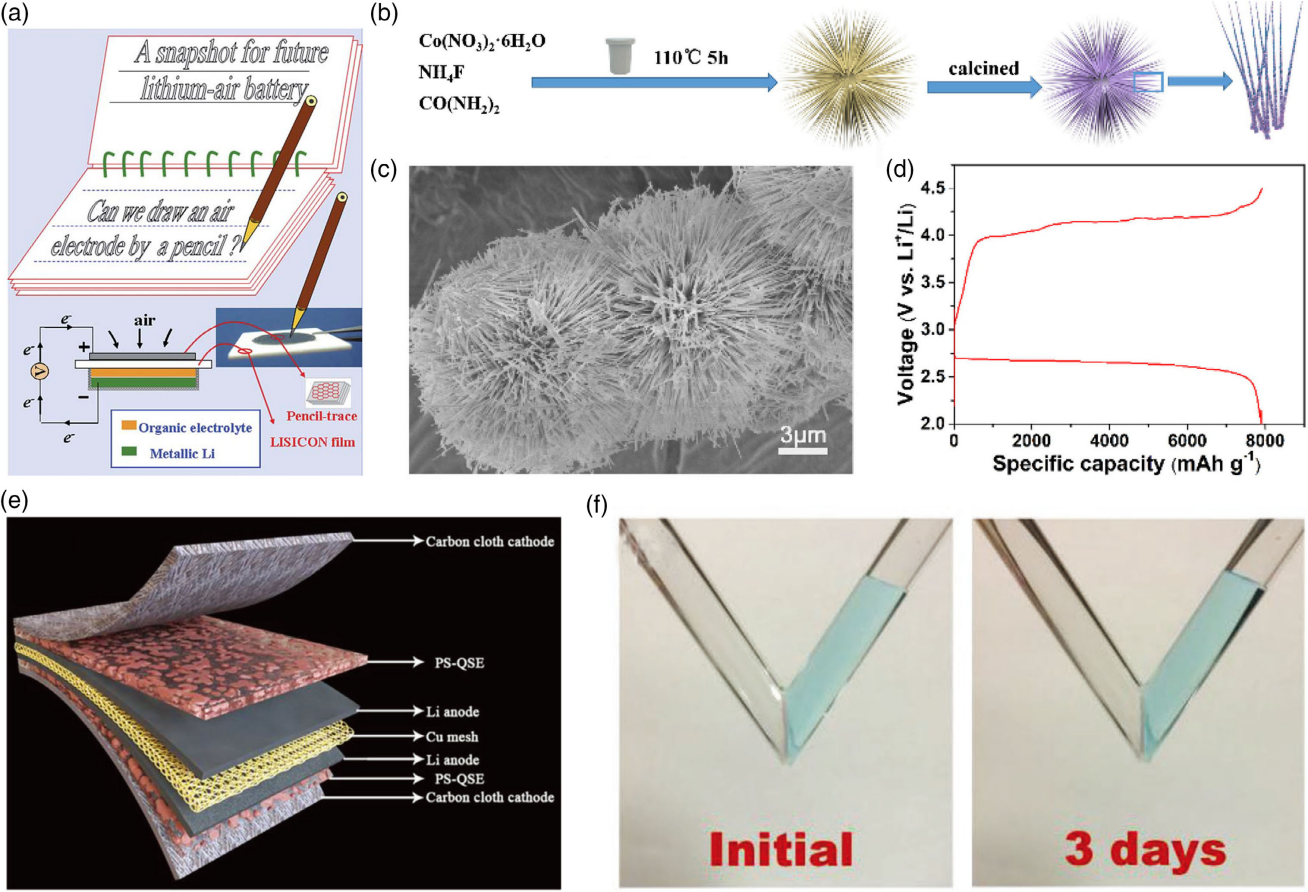
a) Graphical representation of a solid‐state Li−air battery with a pencil‐drawing cathode. Reproduced with permission.^[^ ^48^ ^]^ Copyright 2011, Royal Society of Chemistry. b) Preparation processes of Co_3_O_4_‐NWs. c) SEM image of the obtained Co_3_O_4_ NWs microspheres. d) Discharge/charge curves of the solid‐state Li−O_2_ battery with Co_3_O_4_‐NWs catalyst at 100 mA g^−1^. Reproduced with permission.^[^ ^49^ ^]^ Copyright 2018, Wiley‐VCH. e) Schematic representation of Li−O_2_ batteries based on PS‐QSE. f) H_2_O permeation experiments through the PS‐QSE membrane. Reproduced with permission.^[^ ^51^ ^]^ Copyright 2019, Wiley‐VCH.

As the critical component of solid‐state Li−air battery, air cathode loading with catalysts is responsible for the heart core reaction region, which plays a key role in practical battery performance including the rate capacity, catalysis kinetics, overpotential, discharge capacity, cycling stability, and so on. In essence, the structure and design of the cathode determine the electron conductivity, mass transfer, the rate of oxygen diffusion, and the storage of discharge products. Therefore, the cathode with highly efficient catalysts for superior catalysis activity, well‐designed porous structure for favorable oxygen/Li‐ion transport and adjustable product size, superior conductive support for good electronic transport, sufficient void volume for the storage of generated discharge products is preferable to an idea air cathode for solid‐state Li−air battery. Carbonaceous materials such as carbon nanotubes, graphene, super P owning favorable electronic conductivity, light mass, low cost, large surface area, particular framework are commonly utilized as electronic conductive substrate and catalyst for solid‐state Li−air battery. Furthermore, the favorable modified interface with lower interfacial resistance between carbonaceous material and solid electrolyte can be achieved by simple heat treatment and mechanical processing, which is indispensable for constructing solid‐state Li−air batteries. Sun et al. fabricated a composite porous air cathode with the composition of garnet LLZTO particles, LiTFSI in polypropylene carbonate or polyimide, and Ketjen black, and the prepared cathode was coated on the LLZTO disk.^[^ ^47^ ^]^ The LLZTO particles ensured an ionic conductive framework and space for air diffusion, LiTFSI in polypropylene carbonate as the conductive binder ensured favorable ion conduction at the LLZTO interfaces. The effective porous catalyst is favorable to the Li_2_CO_3_ decomposition and the accommodation of discharge products. The assembled solid‐state Li−air batteries delivered a specific capacity of 20 300 mAh g^−1^ carbon at 20 μA cm^−2^ in open air at 80 °C and operated 50 cycles without capacity fading at a cutoff discharge capacity of 1000 mAh g^−1^_carbon_. Zhou's group constructed an air catalytic electrode by pencil‐drawing with 2D multilayered nanosheet on LISICON solid electrolyte (**Figure** **6**a).^[^ ^48^ ^]^ The fabricated Li−air battery with a solid–liquid composite electrolyte consisting of a LISICON ceramic film and an organic liquid electrolyte, delivered a discharge capacity of 950 mAh g^−1^ at 0.1 A g^−1^.

Similar to the problem with carbonaceous cathode material in nonaqueous Li−air battery, carbon decomposition is caused by the side reactions between carbon and discharge product or intermediate under high potential in solid‐state Li−air batteries.^[6d]^ The exploration of carbon‐free cathode is expected to overcome this disadvantage of carbon materials. Liu et al. developed a solid‐state Li−O_2_ cells with a composite solid electrolyte membrane consisting of LLZO powder and PVDF‐HFP matrix, and a porous catalyst of Co_3_O_4_ nanowires (Co_3_O_4_‐NWs) microspheres (Figure 6b,c).^[^ ^49^ ^]^ The oxygen cathode exhibited abundant catalytic activity sites based on a high specific surface area of the porous Co_3_O_4_‐NWs. A closed initial capacity of 8000 mAh g^−1^ to the battery with liquid electrolyte and a long cycle life of 68 cycles with low polarization was achieved at 100 mA g^−1^ in the assembled solid‐state Li−O_2_ battery (Figure 6d), demonstrating the effective porous Co_3_O_4_ microspheres in solid‐sate Li−O_2_ batteries. Based on the consideration of the contact of the cathode with solid electrolytes for favorable electron and mass transport as well as the abundant exposed active sites, the selective application of other highly efficient carbon‐free materials in nonaqueous Li−O_2_ batteries is promising to solid‐state Li−air batteries.

Furthermore, designing multifunctional catalysts is essential to achieve safe, stable Li−air batteries with performance. For different problems, based on the consideration of the advantages of each material, the multifunctional catalysts would provide an effective method to simultaneously address several problems. Huang et al. reported an integrated graphene‐based quasi‐solid‐state Li–O_2_ battery with porous graphene as cathode, tetrathiafulvalene‐modified gel polymer electrolyte, and porous graphene/Li composite as an anode.^[^ ^50^ ^]^ Tetrathiafulvalene as a redox mediator combined with the 3D porous graphene were utilized to catalysis the battery reaction. The assembled quasi‐solid‐state Li–O_2_ coin cell displayed a stable discharge–charge process of 100 cycles at the cutoff capacity of 2000 mAh g^−1^ with a current density of 1000 mA g^−1^, a low overpotential of 0.95 V, as well as the superior rate performance, demonstrating the effective role of the introduction of tetrathiafulvalene in reducing the charge overpotential and the favorable nanoporous graphene cathode to ensure the fast O_2_ reduction and tetrathiafulvalene oxidation and facilitate transport for O_2_, Li ions, and tetrathiafulvalene, paving a promising avenue for constructing safe, full performance practical Li−air batteries in future.

#### 3.3.2 Li–Metal Anode Optimization

Compared with the nonaqueous electrolyte, solid electrolyte is obviously superior in Li anode protection and thus the safety and stability of Li−air batteries. However, the Li anode is still suffered from the corrosion reaction of the Li anode, the Li dendrites, and volume change during Li plating/stripping, leading to inferior cycling span, and low Coulombic efficiency. First, due to the existence of free volume in some solid electrolyte matrices such as the polymer, H_2_O, and CO_2_ in the air are prone to enter into the side of the Li–metal anode through the solid matrix, resulting in the Li–anode corrosion and poor mechanical stability of the electrode/electrolyte interface. Shu et al. directly coated a membrane consisting of hydrophobic silica and PVDF‐HFP on the Li metal for flexible solid‐state Li−O_2_ battery (Figure 6e), the protection of Li metal by this hydrophobic quasi‐solid membrane (PS‐QSE) was carried out through the H_2_O contact angle and permeation tests (Figure 6f).^[^ ^51^ ^]^ A stable H_2_O contact angle of 113° after 10 min and negligible H_2_O permeation into the membrane demonstrated the favorable protection of the Li anode from being corroded by the crossover of moisture or H_2_O. In addition, in situ formation of passivation or SEI film on Li meal anode, constructing Li alloy anode and the delicate framework design of Li metal anode is also promising to tackle the dendrite formation and volume change issues.^[^ ^52^ ^]^ For instance, Li alloy anode can be obtained by alloying the Li metal with metallic or metalloid elements.^[^ ^53^ ^]^ Hassoun et al. reported a Li‐Si alloy anode by replacing Li metal anode with lithiated silicon‐carbon composite to improve the safety of Li−air battery.^[52b]^ The battery theoretical energy density is up to 980 Wh kg^−1^ according to the mass of cathode and anode. Although this design is proposed in nonaqueous Li−air batteries, it is also applicable to the Li metal anode design of solid‐state Li−air battery. More methods referred to the interfacial regulation are also effective in improving the stability and safety of Li metal, which are illustrated in the later section.

#### 3.3.3 Electrolyte Design

The selection of a favorable solid electrolyte is quite important to the overall solid‐state Li−air battery performance. Ideal solid electrolytes with superior thermostability, high Li‐ion transference number, and negligible electronic conductivity, wide electrochemical window (above 5 V versus Li^+^/Li), excellent mechanical strength, good shape flexibility as well as the excellent interface stability toward electrodes are expected in achieving high‐performance solid‐state Li−air battery with long cycling life and superior safety.

Given the above, the design of solid electrolyte should observe the following aspects: higher Li ionic conductivity and lower electronic conductivity to ensure fast interior kinetics in the solid electrolytes and interfacial kinetics between solid electrolytes and active materials, chemically or electrochemically stable against the reactive oxygen species, protection of cathode or anode from the generation of undesired side products at the electrode/electrolyte interface by preventing the crossover of undesired chemical substances. Superior ionic conductivity is crucial to reaction kinetics. Impurity element doping is considered to be favorable strategy to enhance the ionic conductivity of the inorganic solid electrolyte, in which the doped element can improve the concentration of certain ions and vacancies, as well as the structure stability.^[^ ^54^ ^]^ For the polymer electrolyte, lowering the polymer crystallinity and enhancing its plasticity are necessary to enhance the ionic conductivity, which can be achieved through polymer blends, lowering the structure order by the addition of crosslinking agent for linking up the polymer chain, reducing crystallinity by the addition of plasticizers.^[^ ^55^ ^]^ The commonly utilized plasticizers contain the metal–organic framework, inorganic fillers, organic solvents such as carbonates, ethers and ionic liquids.^[^ ^39^, ^56^ ^]^ Furthermore, the wide electrochemical window of solid polymer determines its feasibility in solid‐state Li−air batteries. When investigating the electrochemical window of polymer electrolytes, both the redox window of the polymer itself and that of the dissolved Li salts or other additives should be taken into account. Additionally, the chemical/electrochemical stability toward cathode/anode and the compatibility with electrodes in the open working environment can be tackled through the interface regulation strategies in the following section.

#### 3.3.4 Interface Regulation Between Electrode and Electrolyte

Aside from the above‐referred cathode, anode, solid electrolyte, the interface regulation between electrode and electrolyte is also critical to the high‐performance solid‐state Li−air battery. High interfacial resistances between cathode and electrolyte or electrolyte and Li metal anode, and chemical instability of certain solid electrolyte toward Li metal or H_2_O, CO_2_ in the open air atmosphere are of critical challenges to be tackled for solid‐state Li−air batteries. An ideal solid electrolyte/electrode interface is expected to be nanometrically thin and buried in the composite electrode, accounting for only a small part of the battery's mass and volume.^[^ ^16^ ^]^ Accordingly, a negligible interfacial resistance together with a stable interface between electrode and solid electrolyte is essential to solid‐state Li−air batteries.

**Figure 7:**
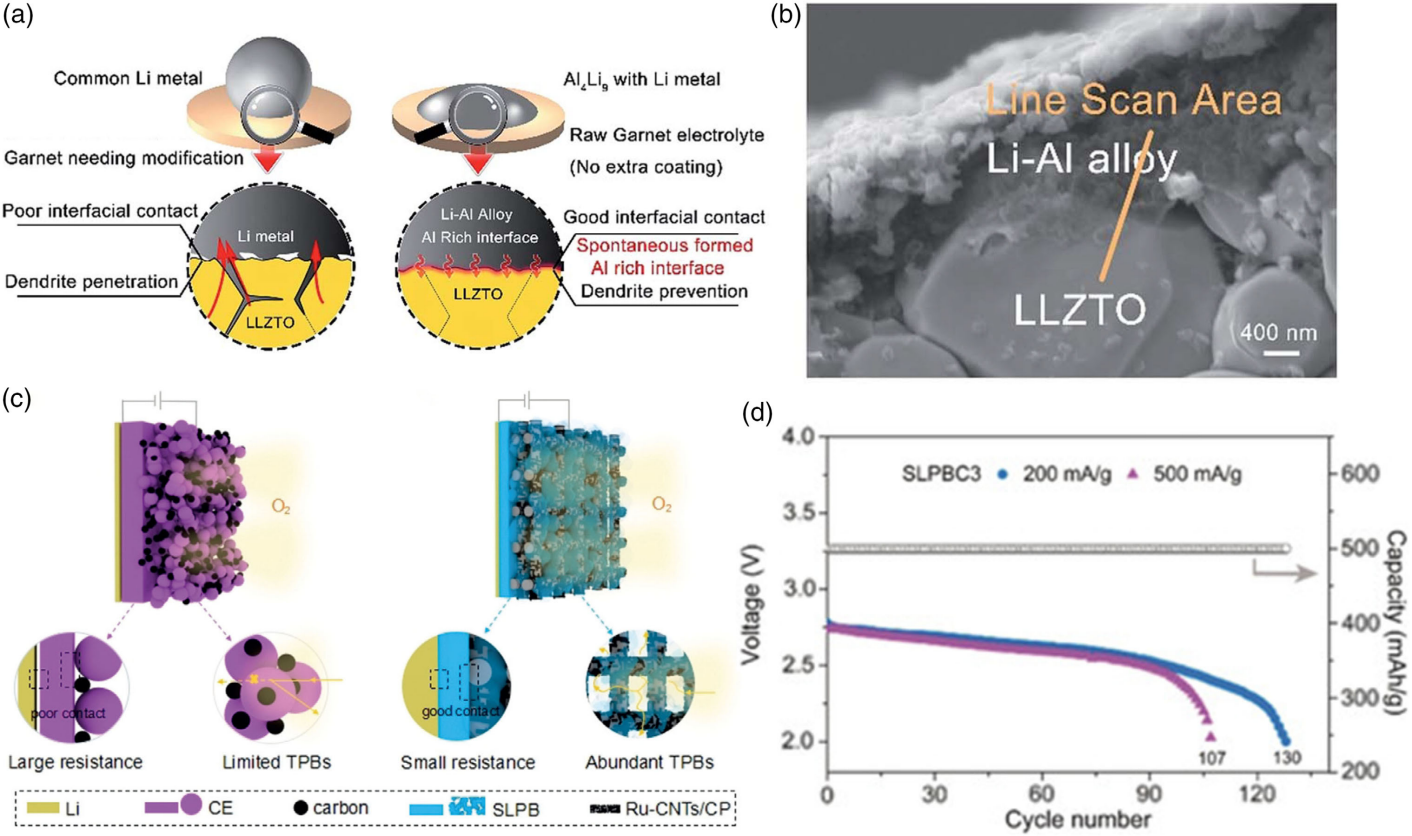
a) Interfacial stability comparison between the common Li‐garnet interface and the Li‐Al alloy induced Al‐rich interface. b) SEM images of LiAl alloy/LLZTO‐LZO interface. Reproduced with permission.^[^ ^59^ ^]^ Copyright 2018, Royal Society of Chemistry. c) Schematic illustration of all‐solid Li−O_2_ battery based on conventional ceramic electrolyte (CE) and the prepared SLPB, in which TPBs indicate triple‐phase boundaries. d) Cycling performance of the all‐solid Li−O_2_ batteries based on SLPB at 500 mAh g^−1^. Reproduced with permission.^[^ ^61^ ^]^ Copyright 2020, Wiley‐VCH.

In comparison with the liquid Li−air battery, the solid‐solid interface makes high interfacial impedance and inferior wettability toward air cathode and Li anode, furthermore, the existence of a large interfacial resistance between the intrinsic low conductivity of the discharge products and solid‐state electrolytes, which directly influence the cycling performance and round‐trip efficiency of Li−air battery. Low interfacial impedance is crucial to a superior solid‐state Li−air battery: 1) First, building a metal or metal oxide coating between solid electrolyte and Li anode provides an effective strategy to reduce the interfacial impedance and improve the interfacial wettability. Wang et al. proposed a simple ZnO coating between the garnet electrolyte and molten Li metal to improve the interfacial wettability, and an ultra‐low interfacial impedance of 20 Ω cm^2^ was achieved.^[^ ^57^ ^]^ Similarly, Han et al. constructed an Al_2_O_3_ coating between Li_7_La_2.75_Ca_0.25_Zr_1.75_Nb_0.25_O_12_ electrolyte and Li anode by utilizing the method of atomic deposition layer, in which the formation of Li–Al–O layer is favorable to lower the interfacial impedance from the initial 1710 to 1 Ω cm^2^ between the anode and solid electrolyte, further the newly formed Li–Al interface is beneficial to the fast Li ionic conductivity, and thus the cycling stability of battery has greatly improved^[^ ^58^ ^]^; 2) Except for the construction of a metal or metal oxide coating, stable SEI construction is another common strategy to achieve lower interface impedance. Wen's group adopted an in situ grown strategy of AlLi metal alloy on the surface of raw garnet‐structured LLZO (LLZTO‐LZO) to build a Li‐rich SEI membrane between the solid electrolyte and Li metal anode with a negligible interface impedance of 1 Ω cm^2^ and a high ionic conductivity of 1.09 × 10^−3^ S cm^−1^ at room temperature.^[^ ^59^ ^]^ Not only is the interfacial wetting improved but also a robust solid‐solid SEI is successfully built (**Figure** **7**a,b). The battery performed a stable cycling of over 3000 h with no sudden voltage drop and stable voltage polarization. He et al. introduced an a Li–Sn alloy layer between solid electrolyte and Li anode, a decreased interfacial resistance by 20 times, and favorable, stable Li ionic conductivity even under high current densities were achieved^[^ ^60^ ^]^; and 3) Last, but definitely not least, is the integrated design of electrolyte and porous cathode to tackle the inferior interfacial contact by introducing solid electrolyte in situ integrated on the surface of the porous cathode, this feasible strategy not only ensure the sufficient and continuous triple‐phase boundaries and superior solid‐solid contact interface, meeting the simultaneous requirements of Li ions and electron transfer and favorable diffusion of gas for a high‐performance Li−air battery. Recently, our group have attempted to introduce an integrated metal–organic framework/reduced graphene oxide aerogel structure by introducing metal–organic framework‐based solid electrolyte layer owning abundant Lewis‐acidic sites and abundant Li‐ion transport channels on porous metal–organic framework/reduced graphene oxide and initial expected results have been achieved. Taking advantage of the unique chemical properties of the metal–organic framework‐based solid‐state electrolyte layer and the integrated battery structure, the solid‐state Li−O_2_ battery displays low‐impedance wettable electrode/electrolyte interfaces, effectively preventing the anode dendrite formation and air corrosion. Zhang's group constructed an all‐solid‐state Li−O_2_ battery with an integrated design by in‐situ introducing porous succinonitrile‐based plastic crystal electrolytes (SLPB) on the surface of the carbon cathode (Figure 7c).^[^ ^61^ ^]^ Combining the superior Li‐ion conductivity, adhesion of thin, soft SLPB with the abundant, continuous triple‐phase boundaries, the assembled all‐solid‐state Li−O_2_ battery displayed based on this integrated design delivered a high discharge capacity of 5963 mAh g^−1^, and a stable cycling life of 130 cycles at 200 mA g^−1^, 500 mAh g^−1^ (Figure 7d). The comparative experiments with pure carbon nanotubes as the cathode further demonstrated the critical role of triple‐phase boundaries on the battery capacity. Zhu et al. constructed an integrated Li−O_2_ battery structure by coating a LATP solid electrolyte on the surface of a porous carbon air cathode mixed with LATP powder. Benefiting from the expanded reaction boundaries of the entire solid‐state cathode from this integrated structure, the Li−O_2_ battery delivered a discharge capacity of 16 800 mAh g^−1^ during the first cycle.^[46a]^ For the large interfacial resistance caused by the insulated solid discharge product and solid electrolyte, combining solid electrolyte with redox mediator would be a novel strategy, in which the oxidized state of the redox mediator is able to react with Li_2_O_2_ to generate Li ions and O_2_, and turns into the initial state. Kim et al. proposed a novel gel polymer electrolyte that is composed of PVDF as the solid electrolyte and *p*‐benzoquinone as the redox mediator.^[^ ^62^ ^]^ With the addition of *p*‐benzoquinone, the interface resistance was decreased, and the Li−O_2_ battery with this gel composite electrolyte performed improved cycling stability in comparison with the single PVDF.

Except for the high interfacial resistances between the electrode and solid electrolyte, the chemical/electrochemical instability is still deeply concerned. The side reactions between the electrode and solid electrolyte, as well as Li dendrites growth, are considered to be two main challenges. Although solid electrolyte shows superior chemical stability compared with nonaqueous electrolyte, unstable drawbacks still exist for some electrolytes such as the NASICON solid electrolyte containing transition metal ions with high oxidation state is prone to react with Li anode, garnet‐type solid electrolytes are sensitive to H_2_O and CO_2_ in ambient air. For example, Ti^4+^ in LATP, Ge^4+^ in LAGP, are prone to occur the reduction reaction with Li metal and form a reaction layer similar to the formation of the SEI layer.^[^ ^63^ ^]^ For that matter, constructing a Li ions‐conducting interlayer or related metal coating between Li anode and solid‐state electrolyte provides a feasible strategy to inhibit the direct contact of electrolyte with Li metal anode and improve the chemical stability of certain solid‐state electrolyte.^[^ ^64^ ^]^ Zhou's group built an amorphous Ge thin film on the surface of LAGP solid electrolyte through the sputtering method, which was utilized as the solid electrolyte of Li−air battery.^[^ ^65^ ^]^ The coated Ge metal film effectively isolated the direct contact with Li anode and thus inhibit the reduction reaction of Ge^4+^. The open Li−air battery based on this Ge^0^ film‐coated LAGP solid electrolyte delivered a discharge capacity of 1000 mAh g^−1^ at 200 mA g^−1^ and retained a stable 30 cycles. For the generation of thick Li_2_CO_3_ layer covered on the surface of the garnet solid electrolyte caused by the side reaction between the garnet‐type solid electrolyte and CO_2_, various surface polishing processes, heat treatment, and recently the carbon treatment is utilized to remove the Li_2_CO_3_ layer from the solid electrolyte.^[^ ^14^, ^34^, ^66^ ^]^ It is generally assumed that the introduction of solid‐state electrolyte could well overcome the challenge of Li dendrites, but in fact, this challenge still exists for some solid‐state electrolyte, especially for the type of garnet‐structured LLZO with high electronic conductivity, Li dendrites are likely to grow along the grain boundary and interconnected pores inside the solid electrolyte, resulted in short circuit and battery failure after several cycles.^[^ ^67^ ^]^ Constructing dense solid electrolyte with little pores and cracks, thermal treatment, thin dense alloying layer on solid electrolyte provide an appropriate solution in inhibiting the growth of Li dendrites.^[^ ^68^ ^]^

In conclusion, building a metal or metal oxide coating between the electrolyte and Li metal, constructing a protected suitable SEI, an integrated design of solid electrolyte with porous cathode by in situ reactions provide effective strategies in achieving low interfacial resistance and enhanced ionic transport. Constructing a Li ions‐conducting interlayer or related metal coating between Li anode and solid‐state electrolyte, adopting essential physical surface treatments are favorable to improved chemical/electrochemical stability for a stable electrolyte/electrolyte interface together with reduced interfacial resistance and depressed Li dendrite growth. The well, rational designed electrode/solid electrolyte interface provides a promising prospect for the practical application of solid‐state Li−air batteries, although the interface regulation of electrode/solid electrolyte remains a quite challenge.

## 4 Future Perspectives and Outlook of Solid‐State Li−Air Batteries

**Figure 8:**
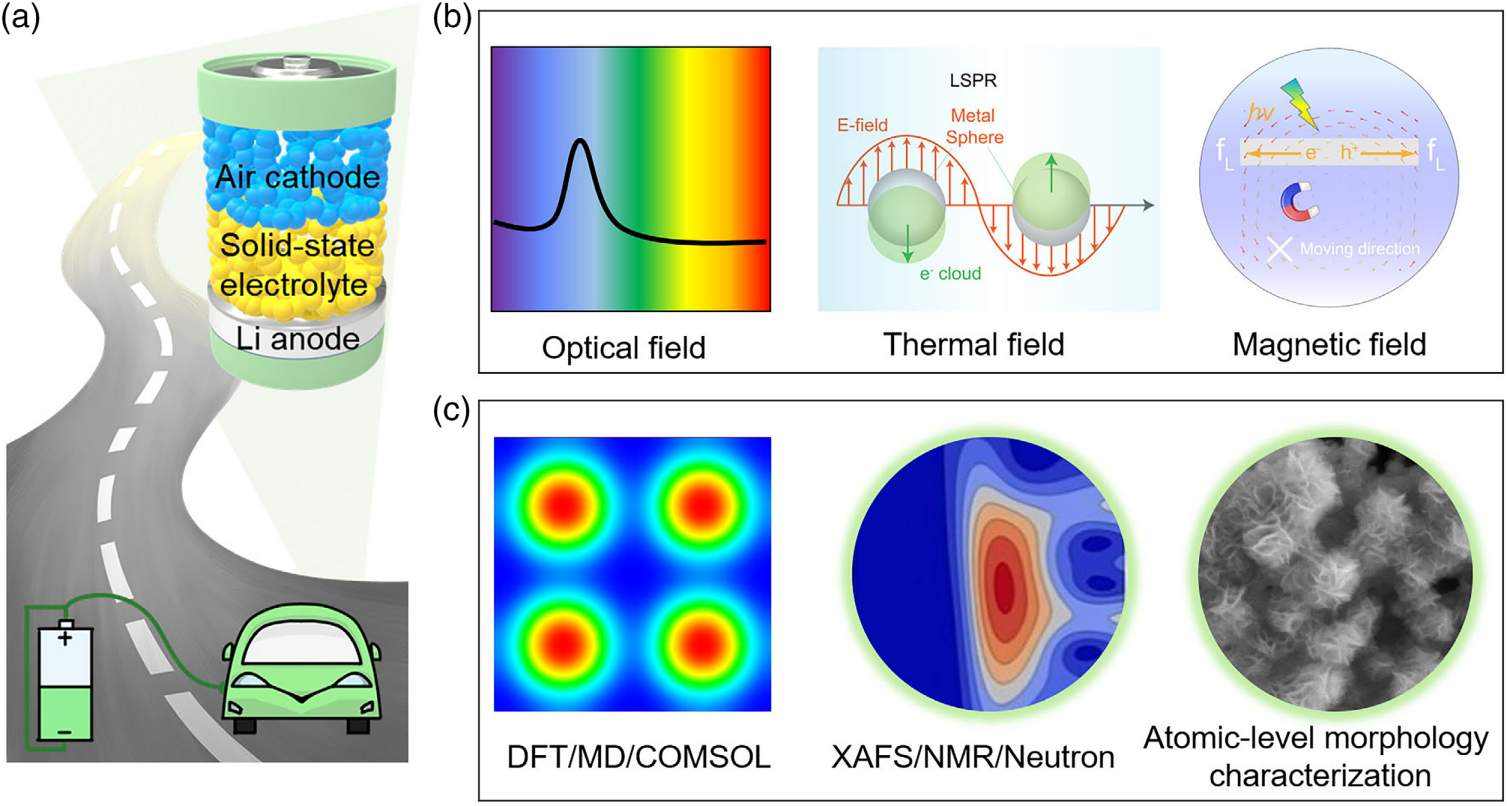
Prospect and outlook for solid‐state Li−air batteries. a) Developing preferable battery materials. b) Exploring novel battery systems. c) In‐depth mechanism study with advanced characterization techniques, in which the density functional theory calculations, molecular dynamics simulations, COMSOL Multiphysics, X‐Ray absorption fine structure analysis, nuclear magnetic resonance, neutron diffraction are shortened to DFT, MD, COMSOL, XAFS, NMR, Neutron, respectively.

Combining all of the aforementioned illustrations, the solid‐state Li−air batteries are considered to be the most promising strategy in eventually achieving safe, stable, high‐performance practical Li−air batteries directly operated in ambient air in the future. Although great progress together with irreplaceable security advantages has been achieved in current solid‐state Li−air batteries, multiple, systematic, integrated research efforts referring to perfect material exploration including electrodes, solid‐state electrolytes, electrode/electrolyte interface additives, the effect of solid electrolyte on discharge products, and the exploration of novel battery system (**Figure** **8**a,b); the ion‐transport mechanisms and the working mechanism of solid‐state Li−air battery from the microscopic view are still indispensable before realizing their commercialized application in the energy system (Figure 8c).

### 4.1 Developing Favorable Battery Materials and Novel Battery Systems

First, well‐designed, multifunctional, porous composite air cathode with superior chemical/electrochemical stability toward oxygen radicals, air components, continuous ions/electron transmission path, and excellent catalysis activity is expected to be explored to achieve high‐performance battery. Second, stable cathode and solid electrolyte substrates combining the superiorities of battery components are expected to be developed to achieve a stable cell structure, such as a non‐carbon support layer on the surface of the carbon frame for air cathode. Furthermore, the in situ passivation or alloy layer on Li metal anode along with a robust cell structure is favorable to inhibiting the Li dendrite growth and volumetric change during long‐term battery cycling. Finally, and most important, developing a preferable electrolyte with superior ionic conductivity, low interfacial impedance, and chemical/electrochemical compatibility toward both the cathode and Li anode is necessary, for example, a thin flexible layer with ionic conductivity or electron conductivity deposited on the surface of the electrode or solid electrolyte, an ionic conductive interlayer between electrolyte and cathode/anode would offer a preferable interface between electrolyte and cathode/anode to overcome the solid–solid interfacial issues.^[6a]^ Further research regarding composite ionogel electrolyte is anticipated. Additionally, the exploration of outfield‐assisted (such as optical field, thermal field, electric field, magnetic field) solid‐state Li−air batteries on the basis of the related specific field effects is also a promising direction in developing applicable advanced battery systems.[^7^, ^69^]

### 4.2 In‐Depth Defined Mechanism Study by Multiple Characterization Techniques

A systematic, in‐depth research with the combination of lab experiments and related theoretical calculation, as well as the advanced characterization techniques to study the definite ion‐transport mechanisms, Li deposition mechanism through solid electrolytes, and the specific interfacial issues between solid electrolyte and electrode materials are crucial to develop optimized component materials and construct an ideal solid electrolyte/electrode interface for practical solid‐state Li−air batteries. Furthermore, the working mechanism of solid‐state Li−air battery is still in its infancy due to the lack of suitable electrolyte, which is more complex in ambient air in comparison with the Li−O_2_ battery. More products on cathode such as LiOH, Li_2_CO_3_ from the reactions with H_2_O, CO_2_ in ambient air would generate during discharge, resulting in complex electrochemical reactions. Therefore, a defined, in‐depth understanding of the battery working mechanism is notably important to eventually achieve advanced open battery systems with reliable safety, high energy density, and long cycling life.

## Conflict of Interest

The authors declare no conflict of interest.
